# Endoplasmic reticulum stress response in an INS-1 pancreatic β-cell line with inducible expression of a folding-deficient proinsulin

**DOI:** 10.1186/1471-2121-11-59

**Published:** 2010-07-26

**Authors:** Taila Hartley, Madura Siva, Elida Lai, Tracy Teodoro, Liling Zhang, Allen Volchuk

**Affiliations:** 1 Division of Cellular and Molecular Biology, Toronto General Research Institute, University Health Network, 101 College Street, TMDT 10-706, Toronto, M5G 1L7, Canada; 2 Department of Biochemistry, University of Toronto, 1 King's College Circle, Medical Sciences Building 5th Floor, Toronto, M5S 1A8, Canada; 3 Department of Physiology, University of Toronto, 1 King's College Circle, Medical Sciences Building 3rd Floor, Toronto, M5S 1A8, Canada; 4 University Health Network, 101 College Street, TMDT 10-706, Toronto, M5G 1L7, Canada

## Abstract

**Background:**

Cells respond to endoplasmic reticulum stress (ER) stress by activating the unfolded protein response. To study the ER stress response in pancreatic β-cells we developed a model system that allows for pathophysiological ER stress based on the Akita mouse. This mouse strain expresses a mutant insulin 2 gene (C96Y), which prevents normal proinsulin folding causing ER stress and eventual β-cell apoptosis. A double-stable pancreatic β-cell line (pTet-ON INS-1) with inducible expression of insulin 2 (C96Y) fused to EGFP was generated to study the ER stress response.

**Results:**

Expression of Ins 2 (C96Y)-EGFP resulted in activation of the ER stress pathways (PERK, IRE1 and ATF6) and caused dilation of the ER. To identify gene expression changes resulting from mutant insulin expression we performed microarray expression profiling and real time PCR experiments. We observed an induction of various ER chaperone, co-chaperone and ER-associated degradation genes after 24 h and an increase in pro-apoptotic genes (*Chop *and *Trib3*) following 48 h of mutant insulin expression. The latter changes occurred at a time when general apoptosis was detected in the cell population, although the relative amount of cell death was low. Inhibiting the proteasome or depleting Herp protein expression increased mutant insulin levels and enhanced cell apoptosis, indicating that ER-associated degradation is maintaining cell survival.

**Conclusions:**

The inducible mutant insulin expressing cell model has allowed for the identification of the ER stress response in β-cells and the repertoire of genes/proteins induced is unique to this cell type. ER-associated degradation is essential in maintaining cell survival in cells expressing mutant insulin. This cell model will be useful for the molecular characterization of ER stress-induced genes.

## Background

An increase in post-prandial blood glucose stimulates pancreatic β-cells to secrete insulin, which mediates glucose disposal into tissues such as muscle and fat. Glucose also acutely stimulates insulin translation, which replenishes the β-cell granules that have been depleted during secretion [1]. The effect of glucose on translation increases the protein folding load in the endoplasmic reticulum (ER) of the β-cell, which may transiently induce ER stress, a condition in which the folding capacity of the ER is insufficient for the level of newly synthesized secretory proteins and results in the accumulation of misfolded proteins. Such conditions are sensed by ER localized stress sensors that mediate the unfolded protein response (UPR) [2]. This results in a transient reduction in translation efficiency to reduce secretory protein load and induces chaperone capacity and ER-associated degradation (ERAD). ER stress is likely a physiological situation the β-cell transiently experiences following feeding.

ER stress is also a feature of pathological conditions associated with obesity and diabetes. Excess nutrients (free fatty acids, glucose), amyloid deposits and inflammatory cytokines have all been shown to induce ER stress in pancreatic β-cells [3]. These conditions induce the UPR pathways that attempt to counteract ER stress. However, chronic ER stress can lead to apoptosis induction [4], particularly in cell types with high secretory capacity such as the β-cell. This is evident from the fact that perturbation of the UPR signaling pathways *in vivo *leads to pancreatic β-cell death in animal models [5-7] and humans [8] and mutations in the insulin molecule in Akita mice results in ER stress-induced β-cell apoptosis [9,10]. In addition, it has been hypothesized that chronic conditions associated with obesity over time may lead to chronic ER stress-induced β-cell death and depletion of β-cell mass that results in type 2 diabetes [11].

Thus, understanding how pancreatic β-cells deal with, and respond to ER stress, is vital for understanding β-cell biology under physiological and pathological conditions. A detailed temporal analysis of the ER stress response in β-cells has not been conducted. In addition the molecular mechanism of ER stress-induced apoptosis is still, for the most part, unclear. As mentioned, expression of folding-deficient proinsulin in the Akita mouse leads to pancreatic β-cell death as a result of ER stress and apoptosis [9]. Thus, the Akita mouse provides a model of β-cell dysfunction resulting from ER stress and affords a system to study the ER stress response independently of using commonly used toxins such as thapsigargin or tunicamycin.

To study the ER stress response in pancreatic β-cells we developed a pancreatic β-cell culture system based on the Akita mouse. Inducible expression of mutant insulin 2 (C96Y) fused to EGFP [Ins2 (C96Y)-EGFP] caused ER stress and apoptosis. In this study we examined the temporal gene expression response resulting from induction of Ins2 (C96Y)-EGFP expression in INS-1 β-cells and the role of ERAD in mediating cell survival.

## Methods

### Cloning of the pTRE-Tight Insulin 2 (C96Y)-EGFP construct

Full-length wild-type mouse insulin 2 cDNA was amplified by PCR from pIns2 (WT)-EGFP, a fusion construct obtained from Dr. Seiichi Oyadomari (New York University School of Medicine) [12]. The PCR fragment was cloned into pCRII-TOPO vector (Invitrogen). Using the QuikChange II XL Site-Directed Mutagenesis kit (Stratagene), a C96Y mutation was introduced into the insulin 2 cDNA. The mutation was confirmed by DNA sequencing. Ins2 (C96Y) was cloned into the pEGFP-N1 vector (BD Biosciences), the stop codon was removed and the resulting fusion construct pIns2 (C96Y)-EGFP was cloned into the NheI/NotI site of a pTRE-Tight vector (Clontech).

### Generation of an inducible Insulin 2 (C96Y)-EGFP expressing INS-1 β-cell line

Initially a stable pTet-ON INS-1 clone was generated by transfecting INS-1 cells (obtained from Dr. Claes Wollheim, University of Geneva) [13] with the pTet-ON plasmid and stable clones were selected following the protocol provided (Clontech). Several clones were isolated and tested by transient transfection using a luciferase reporter assay and the clone with the lowest basal expression and highest doxycyline induction (INS1 pTet-ON #46) was used for generation of the double stable cell line.

Double-stable Ins2 (C96Y)-EGFP expressing cells were generated by co-transfection with a hygromycin resistance plasmid and the pTRE -Ins2 (C96Y)-EGFP plasmid into pTet-ON INS-1 #46 cells and selection by addition of antibiotics: 200 μg/ml geneticin and 50 μg/ml hygromycin B (Invitrogen). Individual clones were isolated and tested for Ins2 (C96Y)-EGFP expression by western blotting and immunofluorescence before and after induction with doxycycline (2 μg/ml) for 24 h. A positive clone was identified and fluorescence-activated cell sorting was used to isolate the highest 20% of EGFP-positive cells in this clonal population after induction with doxycycline for 48 h. The cells were expanded in media without doxycycline and referred to as clone #4S2.

Clone #4S2 cells were maintained in RPMI 1640 (11.1 mM glucose, 1 mM sodium pyruvate, 10 mM HEPES) supplemented with 10% fetal bovine serum, 2 mM L-glutamine, 55 μM β-mercaptoethanol with antibiotics (100 units/ml penicillin and 100 μg/ml streptomycin) and selection drugs (200 μg/ml G418 and 50 μg/ml hygromycin).

### Cell lysis and western blot analysis

Cells were washed in PBS and lysed in ice-cold lysis buffer (1% Triton X-100, 20 mM HEPES, pH 7.4, 100 mM KCl, 2 mM EDTA, 1 mM PMSF, 10 μg/ml leupeptin, and 10 μg/ml aprotinin, 10 mM NaF, 2 mM Na_3_VO_4_, and 10 nM okadaic acid) for 15-20 min on ice. Lysates were centrifuged at 13,000 rpm for 10 min at 4°C and the protein concentration in the supernatant was determined using the BCA protein assay (Pierce). Equal amounts of protein were resolved by SDS-PAGE and immunoblotted as described previously [14]. For detection of insulin and cleaved caspase-3 by immunoblotting, the samples were resolved using 4-12% NuPAGE gels (Invitrogen). The following primary antibodies were used: Phospho-eIF2α (Cell Signaling, #9721), GADD153/CHOP (Santa Cruz, sc-575), KDEL (StressGen, SPA-827), insulin (Santa Cruz, sc-9168), insulin (Dako, A0564), γ-tubulin (Sigma, T6557), polyclonal anti-GFP (obtained from Dr. James E. Rothman, Yale University), monoclonal anti-GFP (Clontech, 632381), cleaved caspase 3 (Cell Signaling, #9661S), ubiquitin (Dako, Z0458), GM130 (Transduction Laboratories, G65120), Hsp90 (Transduction Laboratories, 610418). The polyclonal Herp antibody was provided by Dr. Linda Hendershot (St. Jude Children's Hospital, Memphis, TN).

### Fluorescence microscopy

Cells were treated as described in the figure legends, washed with PBS and fixed with 4% paraformaldehyde in PBS for 20 minutes, washed with PBS and mounted on glass coverslips using Fluoromount G (EM Sciences). GFP fluorescence was visualized with a Zeiss laser scanning confocal microscope. Clone #4S2 INS-1 cells were treated as described in the figure legend, washed with PBS and fixed with 4% paraformaldehyde in PBS for 20 minutes. The samples were processed for immunofluorescence microscopy as reported previously [15] and immunostained with anti-ATF6 (TO13/14) antibody at a 1:100 dilution [14]. Quantitation of ATF6 staining in the nucleus (marked by DAPI staining) was measured using ImageJ Software. The average intensity in arbitrary units in the nuclear area was determined. A minimum of 18 cells for all experimental condition were quantified and the mean relative nuclear intensity is reported.

### Cell Fractionation

Following doxycycline treatment for 72 hours, clone #4S2 cells were washed with PBS and resuspended in homogenization buffer: (0.25 M Sucrose, 4 mM HEPES, 1 mM MgCl_2_, 1.5 mM EDTA containing a complete protease inhibitor tablet (Roche), 1 mM PMSF, 10 mM NaF, 2 mM Na_3_VO_4_, 10 nM okadaic acid). Cells were homogenized on ice with 12 strokes using a ball-bearing cell homogenizer. The resulting homogenate was either centrifuged at 100 000×g for 1.5 h to generate membrane (pellet) and cytosol (SN) fractions, or subjected to a 0.45 M to 2 M sucrose gradient for 18 h at 30 000 rpm, 4°C as outlined in [16]. After ultracentrifugation, 0.5 ml fractions were taken from the top of the gradient.

### Electron Microscopy

Clone #4S2 cells were fixed in 2% glutaraldehyde (EM Sciences) in 0.15 M Sorensen's Phosphate Buffer (pH 7.4) (EM Sciences) for 30 min at room temperature. The cells were washed twice with PBS and collected into new tubes. Samples were prepared for transmission electron microscopy by the electron microscopy facility at Mount Sinai Hospital (Toronto). Briefly, the cell pellets were fixed in 2% glutaraldehyde in 0.1 M sodium cacodylate buffer (pH 7.3) for 2 h, rinsed with the same buffer for 10 min, and post-fixed for 1.5 h in 1% OsO_4_. After rinsing with the same buffer for 10 min, the cells were dehydrated through a graded ethanol series up to 100% ethanol. The cells were then embedded in Spurr resin in an oven at 65°C overnight. Thin sections (~100 nm) were cut with an RMC MT6000 ultramicrotome. The sections were placed on copper grids and stained with uranyl acetate (20 min) and lead citrate (10 min). The grids were examined in a FEI Tecnai 20 transmission electron microscope and images were captured using a Gatan Dualview digital camera.

### RNA isolation and real-time PCR analysis

For real-time PCR analysis, total RNA was isolated from cells untreated or treated with 2 μg/ml doxycycline for the indicated times using TRIzol Reagent (Invitrogen) followed by purification with RNeasy Mini Kit (Qiagen). Total RNA was reverse transcribed to single-stranded cDNA using the High-Capacity cDNA Reverse Transcription Kit (Applied Biosystems). The resulting cDNA was used for real-time PCR analysis by the TaqMan Gene Expression system (Applied Biosystems) as described previously [14]. The following primers were obtained from Applied Biosystems: *Chop *(Rn00492098_g1), *Atf4 *(Rn00824644_g1), *Grp78 *(Rn00565250_m1), *Pdi *(Rn00564459_m1), *Ero1α *(Rn00593473_m1), *Ero1β *(Rn01520960_m1), *Sel1 *(Rn00710081_m1), *Erdj4 *(Rn00562259_m1), *Erdj5 *(Rn01486444_m1), β-actin (4352931E).

### XBP1 splicing assay

Total RNA was isolated and rat XBP-1 cDNA was amplified by RT-PCR (QIAGEN OneStep RT-PCR kit) using primers that flank the intron excised by IRE1 exonuclease activity as described previously [14].

### siRNA transfection

Clone #4S2 cells were reverse transfected using Lipofectamine RNAiMAX with 10 nM Herp siRNA (Invitrogen) or a control siRNA (directed to bacterial β-galactosidase) as described previously [17]. Twenty-four hours later the cells were incubated or not with doxycycline for 48 h then either lysed for western blot analysis or for measurement of apoptosis using the cell death ELISA kit (Roche).

### Apoptosis assays

Cell apoptosis was measured using the cell death detection ELISA kit (Roche) according to the instructions provided in the kit. Briefly, clone #4S2 cells were seeded in 24-well plates (200 000 cells/well) and treated as indicated in the figure legends. Following the treatments, the cells were lysed and oligonucleosomes in the cytoplasm (indicative of apoptosis-associated DNA degradation) were quantified according to the manufacturer's instructions.

Terminal deoxynucleotidyl transferase-mediated dUTP nick-end labeling (TUNEL) assays were performed using the APO-BrdU TUNEL Assay Kit (Invitrogen). Clone #4S2 cells were treated as described in the figure legend, washed with warm PBS, trypsinized, then stained with Fluor-647 dye-labeled anti-BrdU antibody, as described by the manufacturer's instructions. After staining, the samples were analyzed in a FACSCalibur flow cytometer (Becton Dickinson). To standardize each sample and set the quandrants, a negative control (INS-1 cells) and a positive control (provided by the APO-BrdU TUNEL assay kit) were used. GFP and the Fluor-647 dye-labeled anti-BrdU antibody were excited using the FL1 (530/30) and FL4 (661/16) lasers, respectively.

### Microarray analysis

Microarray expression profiling was used to assess the global transcriptional changes in response to Ins2 (C96Y)-EGFP expression in clone #4S2 cells. Cells were induced or not with doxycycline for 24 h, 48 h or 5 days and total RNA was isolated using TRIzol Reagent (Invitrogen) followed by isolation using an RNeasy Mini Kit (Qiagen). Assessment of RNA quality and microarray analysis was performed at the University Health Network (Toronto) microarray centre. Briefly, following RNA quality assessment with an Agilent BioAnalyzer, samples were reverse transcribed to cDNA. cDNA was purified with a cDNA purification module from Affymetrix. Biotin was incorporated during *in vitro *transcription and purified cRNA was then fragmented with a chemical reaction. 15 μg of labeled and fragmented cRNA was hybridized to Rat Genome 230 2.0 arrays (Affymetrix Genechip) for 17 h at 45°C at 60 rpm. The arrays were stained and washed using fluidic stations with antibody and streptavidin phycoerythrin conjugate solutions. All arrays are scanned using an Affymetrix 3000 7G scanner. The data sets were analyzed using GeneSpring software (Version 7.1) (Agilent Technologies) and statistical analysis was performed using GeneSpring software according to the manufacturer's specifications. Genes with a minimum 2-fold difference between control and doxycycline-treated cells in at least two independent experiments at 24 h, 48 h and 5 days are listed in the Additional file 1 and Additional file 2. The raw microarray data has been deposited in the Gene Expression Omnibus (GEO) database (GSE22537).

### Data analysis

Results are presented as mean ± SE. Statistical significance between two experimental conditions was analyzed using a two-sample *t*-test assuming equal variance. Data from several or more groups was analyzed by ANOVA, followed by Tukey post hoc test. P < 0.05 was considered statistically significant.

## Results

### Generation and characterization of the Insulin 2 (C96Y)-EGFP stable INS-1 cell line

**Figure 1:**
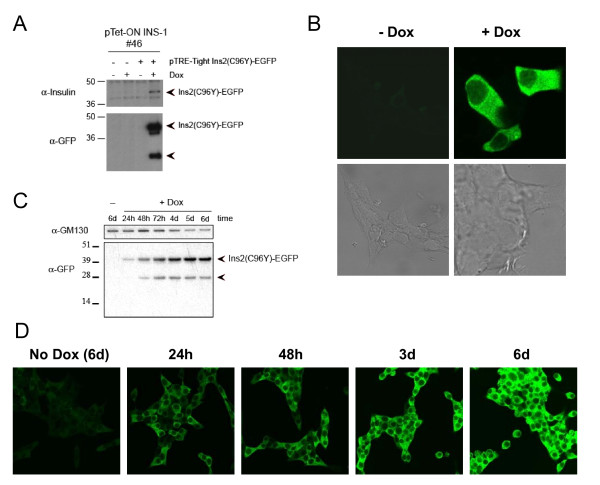
**Induction of insulin 2 (C96Y)-EGFP protein expression in pTet-ON INS-1 cells by doxycyline**. **A-B**. pTet-ON INS-1 #46 cells were transiently transfected with pTRE-Tight Ins2 (C96Y)-EGFP construct in the presence or absence of 2 μg/ml doxycycline (Dox) for 48 h. In (A), cells were washed in PBS, lysed and 10 μg of protein were resolved by SDS-PAGE and immunoblotted using antibodies to insulin and GFP. In (B) the cells were washed in PBS, fixed and mounted. GFP fluorescence was visualized using a laser confocal fluorescence microscope. **C-D**. A stable Ins2 (C96Y)-EGFP expressing INS1 clone (Clone #4S2), generated as described in the methods, was untreated (-Dox) or treated with 2 μg/ml doxycycline (+Dox) for the times indicated. The cells were washed in PBS, lysed and an equal amount of protein per condition were resolved by SDS-PAGE and immunoblotted using antibodies to GM130 and GFP (C). In (D), EGFP fluorescence in fixed cells was visualized by confocal fluorescence microscopy.

We first generated a stable pTet-ON INS-1 cell line that allows for doxycycline-inducible protein expression. The cell line was tested by transient transfection of the Ins2 (C96Y)-EGFP fusion construct in the presence or absence of doxycycline. Fusion protein expression was monitored by western blotting and fluorescence microscopy. Expression of the fusion protein was doxycyline-dependent and the fusion protein was excluded from the nucleus and localized throughout the cell (Figure 1A, B). Interestingly, the expression of this construct also resulted in the production of a lower migrating band, which may be due to proteolytic cleavage of the fusion construct in the cell (Figure 1A, **arrow**). A stable Ins2 (C96Y)-EGFP pTet-ON cell line was generated by co-transfection with a hygromycin resistance plasmid. We isolated a clone with inducible mutant insulin expression and fluorescence-activated cell sorting was used to isolate EGFP-positive cells (top 20%) after 48 h of doxycycline induction. The sorted cells (designated clone #4S2) was expanded in media without doxycycline. This clone exhibited low basal Ins2(C96Y)-EGFP expression and a marked induction following doxycycline treatment (Figure 1C, D).

**Figure 2:**
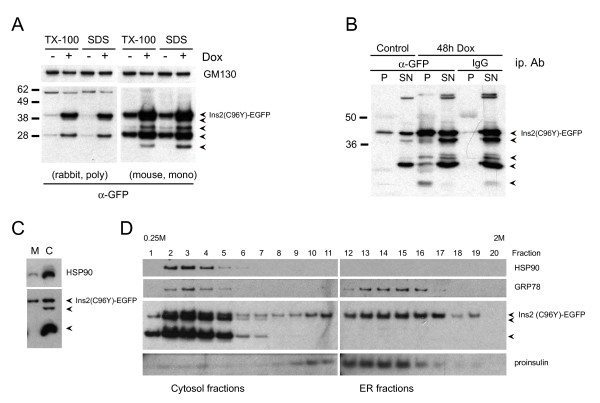
**Insulin 2 (C96Y)-EGFP is localized to the ER while degradation products appear in the cytosol**. **A**. Clone #4S2 cells were untreated (-Dox) or treated with 2 μg/ml doxycycline (+Dox) for 48 h. Following the treatments, the cells were washed in PBS and lysed in either 1% TX-100 lysis buffer (with protease inhibitors) or directly in SDS sample buffer with β-mercaptoethanol. Equal amounts of protein (or equal volumes) were resolved by SDS-PAGE and immunoblotted using antibodies to GM130 or GFP. **B**. Cells were untreated (Control) or treated for 48 h with 2 μg/ml doxycycline before lysis and immunoprecipitation with anti-GFP or control mouse IgG antibodies. The entire precipitate (pellets; P) and 10 μg of the supernatant (SN) were resolved by SDS-PAGE and immunoblotted using an anti-GFP (rabbit, polyclonal) antibody. **C**. Cells were treated with 2 μg/ml doxycycline for 72 h, washed in PBS and homogenized in sucrose buffer as described in the methods. The homogenate was fractionated into membrane (M) and cytosol (C) fractions. **D**. Cells were treated with 2 μg/ml doxycycline for 72 h, washed in PBS and homogenized in sucrose buffer as described in the methods. The homogenate was fractionated on a linear sucrose density gradient and an equal volume of each fraction was resolved by NuPAGE and immunoblotted using antibodies to HSP90, GRP78, GFP and insulin.

In addition to the full-length fusion protein other GFP immunoreactive bands were observed in both TX-100 detergent whole cell lysates or lysates prepared by direct lysis of the cells in SDS sample buffer (Figure 2A). These fragments are likely degradation products of the full-length fusion protein and most of the fragments could not be efficiently immunoprecipitated using an anti-GFP antibody (Figure 2B). Cells were homogenized and subjected to membrane and cytosol fractionation. Whereas the full-length Ins2 (C96Y)-EGFP fusion protein could be detected in the membrane fraction, the degradation fragments were present exclusively in the cytosol fraction (Figure 2C). The full-length fusion protein was detected in ER fractions by sucrose density fractionation while the lower migrating degradation fragments were present exclusively in the cytosol fractions (Figure 2D). Since no degradation fragments were present in microsome fractions it is likely that degradation of the full-length fusion protein occurs in the cytosol. The presence of some soluble ER lumenal GRP78 (and Ins2 (C96Y)-EGFP) in cytosolic fractions in the sucrose gradient may be due to some leakage during cell homogenization.

**Figure 3:**
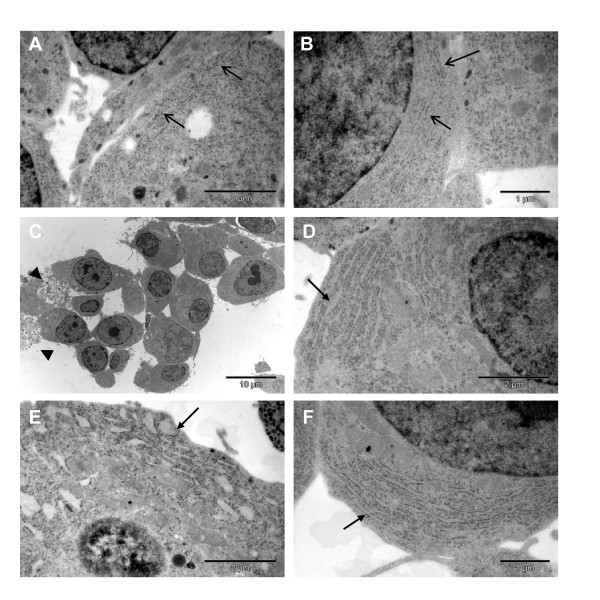
**Effect of insulin 2 (C96Y)-EGFP expression on ER morphology**. Clone #4S2 cells untreated (A, B) or treated with 2 μg/ml doxycycline for 3 days (C, D) or 6 days (E, F) were washed in PBS and fixed. The cells were processed for transmission electron microscopy as described in the methods. ER structures in uninduced cells (A, B, arrows), and distended ER structures in mutant insulin expressing cells following doxycyline-induction (D-F, arrows) are indicated. The presence of apoptotic cells was evident in doxycyline-induced cells (C, arrowheads).

We examined the morphology of the stable cell line by electron microscopy before and after doxycycline induction. In non-induced cells a normal ER morphology was observed (Figure 3A, B), whereas cells expressing Ins2 (C96Y)-EGFP for three days had an altered ER morphology (Figure 3C, D). The ER was expanded and dilated compared to control cells. The ER tended to be even more severely dilated in many of the cells after six days of mutant insulin expression (Figure 3E, F). This morphology is characteristic of unfolded protein accumulation and ER stress [2] and has been observed in β-cells of the Akita mouse [9]. In addition, apoptotic cells were observed in the population expressing the fusion protein (Figure 3C, arrowheads).

### The ER stress response to Insulin 2 (C96Y)-EGFP expression

**Figure 4:**
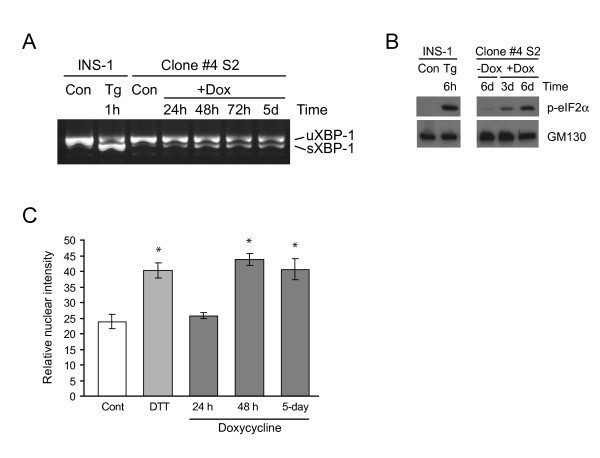
**ER stress signaling pathways are activated by insulin 2 (C96Y)-EGFP expression**. Clone #4S2 cells were untreated (-Dox) or treated with 2 μg/ml doxycycline (+Dox) for the times indicated. Control INS-1 cells exposed to thapsigargin (Tg) or dithiothreitol (DTT) were used as a positive control. **A**. RNA was isolated from the cells and XBP-1 cDNA was amplified by RT-PCR. The unspliced form of XBP-1 (uXBP-1, 480 bp) and spliced form of XBP-1 (sXBP-1, 454 bp) are indicated. **B**. Clone #4S2 cells were treated as in (A), washed in PBS and lysed. Equal amounts of protein were resolved by SDS-PAGE and immunoblotted with anti-phospho-eIF2α and GM130 antibodies. **C**. Clone #4S2 cells were treated with 1 mM dithiothreitol (DTT) for 30 min. or doxycycline as indicated and fixed for immunofluorescence labeling with anti-ATF6 antibody. Nuclear fluorescence of ATF6 was quantified as described in the methods.

The morphological analysis indicated that the expression of Ins2 (C96Y)-EGFP was causing an accumulation of this protein in the ER and causing ER stress. We thus examined whether doxycycline-induced expression of Ins2 (C96Y)-EGFP was activating UPR signaling pathways. Ins2 (C96Y)-EGFP expression was induced for various times and activation of the ER stress-sensing pathways was monitored. Spliced XBP-1, indicative of IRE1α activation, was detected after 24 h of doxycyline induction and similar levels of spliced XBP-1 were present throughout the induction period for up to 7 days (Figure 4A, shows a representative example up to 5 days of induction). The levels of phospho-eIF2α, indicative of PERK activation, were also increased (Figure 4B), as were the levels of active ATF6-p50 (Figure 4C). Thus, expression of the mutant insulin causes measurable activation of the ER stress pathways mediated by IRE1α, PERK and ATF6.

To examine the global transcriptional response to mutant insulin expression we performed microarray analysis of the gene expression changes following Ins2 (C96Y)-EGFP expression. Total RNA was prepared after 24 h, 48 h and 5 days of doxycycline treatment from three independent experiments, reverse transcribed and hybridized to Affymetrix rat genome arrays. Gene expression changes were examined and those that were upregulated or downregulated by 2-fold after doxycyline-induced expression of Ins2 (C96Y)-EGFP are listed in the Additional files 1 and 2. Following 24 h induction 45 genes were upregulated and 5 were downregulated. After 48 h induction 86 genes were upregulated and 37 downregulated, whereas 68 genes were induced and 56 reduced after 5 days induction. These numbers include genes (both well-substantiated and transcribed loci) that were identified as statistically significant in at least two of three experiments.

Shown in Table 1 are a selected list of genes induced >2-fold in at least two independent experiments at the various time points. This list includes genes involved in the UPR response, protein folding and modification, ERAD and protein transport. Several patterns emerged from this analysis. After 24 h of mutant protein expression, the majority of the genes induced were ER localized chaperone genes (*Erdj4/Dnajb9*, *P58^IPK ^*/*Dnajc3*, *Erdj3/Dnajb11*, *Fkbp11*) as well as the ERAD-associated genes (*Herp *and *Sel1*). The *Sdf2l1 *gene was the most abundantly induced gene present in all three experiments. In addition, the *Atf4 *and *Chop *transcription factors, target genes of the PERK-eIF2α pathway, were also induced in two out of three experiments at this early time point. *Trib3 (Trb3)*, a putative target gene of the CHOP transcription factor [18], was also induced in two out of three experiments.

**Table 1:** Selected list of induced genes comparing uninduced cells to cells expressing insulin 2 (C96Y)-EGFP for 24 h, 48 h and 5 days identified by Affymetrix rat genome arrays.

Gene Name	Entrez Gene ID	Putative Function	Fold Change		
			**24 h**	**48 h**	**5d**

**Protein modification/folding**					
*Sdf2l1*	680945	O-mannosylation?	5.7	4.7	10.9
*Fkbp11*	300211	Peptidyl-prolyl isomerase	2.9	3.9	4.3
*Dnajb9/Erdj4*	24908	Protein folding	2.5	3	3.1
*Dnajc3/P58*^*IPK*^	63880	Protein folding	2.3	2.8	4.2
*Dnajb11/Erdj3*	360734	Protein folding	**2.2**	2.4	3
*Pdia2*	287164	PDI family, isomerase		3.1	2.7
*Pdia3/Erp57*	29468	PDI family, isomerase		**3.2**	**3.2**
*Pdia4/Erp72*	116598	PDI family, isomerase		3.1	3.1
*Hspa5/Grp78*	25617	ER Protein folding			2.6
*Pdia6/P5*	286906	PDI family, isomerase			**3**
*Txndc4/Erp44*	298066	PDI family, isomerase			**2**
**ER stress-inducible**					
*Herp*	85430	ERAD	3.3	4.3	**3.5**
*Sel1h*	314352	ERAD	***2.5***	*2.7*	***2.6***
*Atf4*	79255	Transcription factor	**2.7**	3	
*Chop/Gadd153*	29467	Transcription factor	**2.5**	3.5	**2.7**
*Trib3/Trb3*	246273		***11.5***	*19.8*	***6.7***
**Transcription factors**					
*Mist1*	25334	Transcription factor	**2.9**	3.2	**3.7**
*Atf5*	282840	Transcription factor	**2.4**	3.3	
**Protein transport**					
*Kdelr3*	315131	Vesicle transport	**2.1**	2.9	**3.8**
*Tmed3*	300888	Vesicle transport		2.4	2.8
**Selected others**					
*Creld2*	362978	ER Protein folding?	3.6	3.6	7
*Armet*	315989		2.8	2.8	3.9
*Myo1g*	289785	Motor protein	2.3	2.9	**2.5**
*Ell3*	296102	RNA polymerase II-like 3	2.1	2.2	**3.6**
*Magt1*	116967	Magnesium transporter	2.1	**2.3**	2.4
*Oat*	64313	Ornithine aminotransferase	**2**	2.3	2.8

Mean fold change from three independent experiments for each time point is shown. Bold indicates the gene was induced in at least 2 independent experiments and italics indicates average induction value of multiple probes for the same gene.

Following 48 h of mutant protein expression a greater number of genes were induced compared to 24 h (Table 1 and Additional files 1 and 2). Additional chaperone genes (*Pdia2*, *Pdia3 *and *Pdia4*) were upregulated. The levels of the *Chop *transcription factor were also significantly higher than at the 24 h time point. However, the most abundantly induced gene at 48 h of mutant protein expression was *Trib3 *(*Trb3*), a gene whose protein product has been implicated in apoptosis among other cellular processes [18,19]. At this time point other genes including protein transport genes and transcription factors were also upregulated. By 5 days of mutant protein expression the gene expression changes as monitored by microarray was more variable between experiments than at the 48 h time point. However, all the chaperone genes observed at earlier time points were still elevated and additional PDI family member genes (*Pdia6 *and *Txndc4*) were induced. The levels of *Trib3 *were lower compared to the 48 h time point, but still elevated. Interestingly, the levels of *Sdf2l1*, *Creld2 *and *Armet *remained highly induced throughout the time course of mutant protein expression.

**Figure 5:**
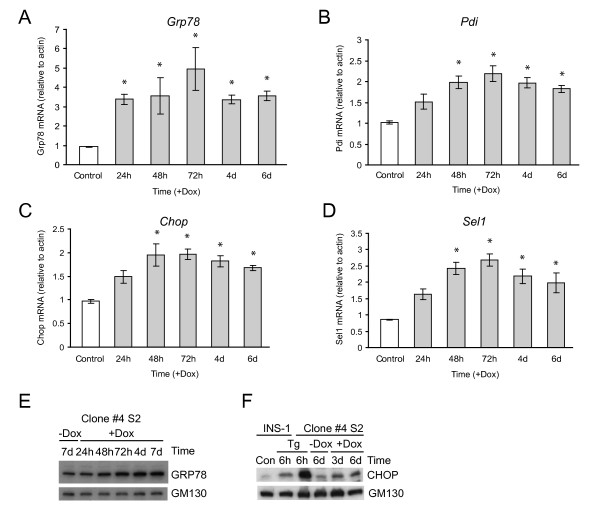
**Expression of insulin 2 (C96Y)-EGFP induces the mRNA and protein of known ER stress response genes**. Clone #4S2 cells were untreated (Control) or treated with 2 μg/ml doxycycline (+Dox) for the times indicated (h, hours; d, days). **A-D**. Total RNA was isolated and the mRNA levels relative to cellular β-actin for *Grp78 *(A), *Pdi *(B), *Chop *(C), *Sel1 *(D) were analyzed by real-time PCR as outlined in the methods. Shown are the mean ± SE of 3 independent experiments. *p < 0.05 relative to control. **E, F**. Clone #4S2 cells were treated as described above, washed in PBS, lysed and equal amounts of protein were resolved by SDS-PAGE and immunoblotted as indicated. Control INS-1 cells exposed to thapsigargin (Tg) were used as a positive control in (F).

Although some of the gene expression changes found were expected based on the ER stress response induced in other models (discussed later), some genes predicted to be increased were not observed, such as the ER chaperone genes *Grp78 *and *Pdi*. Microarray expression profiling can lead to false negatives due to inefficient probes. We therefore examined the mRNA and protein levels of several well established targets of the UPR by real-time PCR and western blotting. An increase in the mRNA levels of *Grp78*, *Pdi*, *Chop *and *Sel1 *was observed in response to doxycycline-induced mutant insulin expression (Figure 5A-D). The latter two changes substantiate the microarray results. However, both *Grp78 *and *Pdi *are significantly upregulated early (within 24-48 h) of mutant insulin expression. This was not detected by microarray, which does produce false negative results in this case. Consistent with the gene expression data, GRP78 and CHOP protein levels were also induced by Ins2 (C96Y)-EGFP expression (Figure 5E-F). Interestingly, thapsigargin treatment for 6 h induced higher expression of CHOP protein in clone #4S2 than in parental INS-1 cells, suggesting that clone #4S2 are more susceptible to ER stress (Figure 5F).

**Figure 6:**
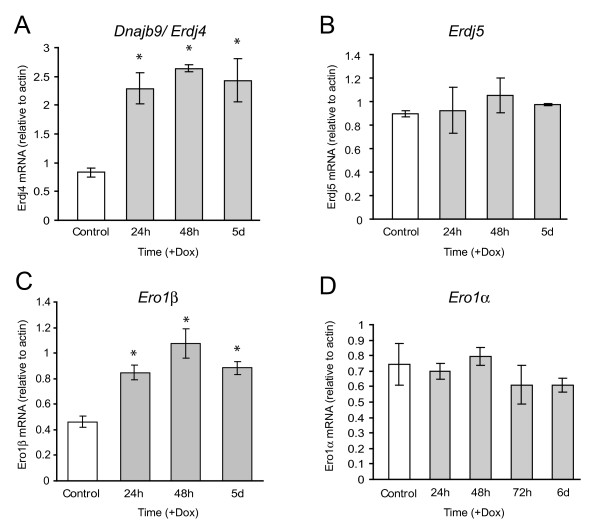
**Expression of insulin 2(C96Y)-EGFP results in the induction of selected genes**. Clone #4S2 cells were untreated (Control) or treated with 2 μg/ml doxycycline (+Dox) for the times indicated. Total RNA was isolated and the mRNA levels relative to cellular β-actin for *Erdj4/Dnajb9 *(A), *Erdj5/Dnajc10 *(B), *Ero1β *(C), *Ero1α *(D) were analyzed by real-time PCR as outlined in the methods. Shown are the mean ± SE of 3 independent experiments. *p < 0.05 relative to control.

We performed additional real-time PCR analysis of other putative ER stress response genes to validate some of the microarray results (Figure 6). Consistent with the microarray data (Table 1) we observed an induction of *Erdj4*, but not the related gene *Erdj5/Dnajc10*, despite the fact that the latter has been shown to be induced by ER stress in other cell types [20,21]. In addition, although not detected by microarray, we observed induction of *Ero1β*, but not *Ero1α *(Figure 6C, D). The former, but not the latter, has been reported to be ER stress-inducible, which is consistent with our results [22]. It is important to note that doxycyline at the same concentration used in these studies added to a parental INS-1 cell line does not induce any changes in *Grp78 *or *Chop *mRNA or protein levels (results not shown), indicating that the changes observed are due to Ins2 (C96Y)-EGFP expression and not the small molecule.

Overall, the microarray and real-time PCR results show that Ins2 (C96Y)-EGFP expression causes the induction of many established ER stress response genes, although there is clearly some selectivity in the genes induced.

### Induction of apoptosis following Insulin 2 (C96Y)-EGFP expression

**Figure 7:**
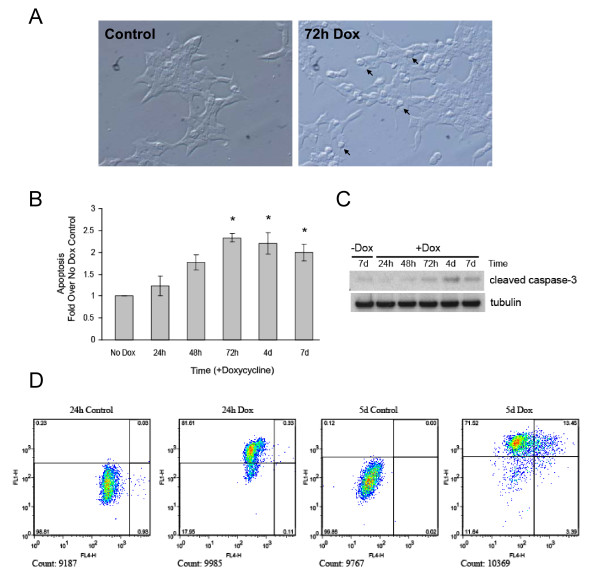
**Expression of insulin 2 (C96Y)-EGFP induces apoptosis**. **A**. Clone #4S2 cells were treated or not with 2 μg/ml doxycycline for 72 h, fixed and imaged by differential interference contrast microscopy. Potentially apoptotic cells with a rounded morphology are indicated (arrows). **B-D**. Clone #4S2 cells were treated with 2 μg/ml doxycycline for the indicated times. **B**. Following the treatments, the cells were lysed and apoptosis was measured using a cell death detection ELISA kit (Roche) as described in the methods. Shown are the mean ± SE of 4 independent experiments. *p < 0.05. **C**. Cells were washed in PBS, lysed and equal amounts of protein were resolved by NuPAGE and immunoblotted using antibodies to cleaved caspase-3 and γ-tubulin. **D**. Cells were fixed and labeled with Alexa Fluor 647 dye-labeled anti-BrdU antibody. Cells were analyzed by flow cytometry; the FL1 laser excites GFP, whereas FL4 excites Fluor 647. Cells in the upper left and right quadrants were classified as mutant insulin expressing, cells in the upper and lower right quadrants were classified as apoptotic. The total number of cells examined is shown below each chart, and the percentage of cells in each quadrant is indicated.

As shown in Table 1, genes associated with apoptosis such as *Chop *and *Trib3 *were already upregulated after 24 h of mutant insulin expression and the expression levels were even greater after 48 h. We thus examined whether doxycycline-induced expression of Ins2 (C96Y)-EGFP induces apoptosis. By light microscopy many of the cells appear rounded in comparison to the control cells at about 72 h of mutant protein expression, indicative of dead or dying cells (Figure 7A, arrows). To confirm that cell death was due to apoptosis we measured cytoplasmic DNA-associated histone complexes using a sensitive ELISA assay and found that 48 h to 7-day treatment of doxycycline causes apoptosis in a time-dependent manner (Figure 7B). Western blot analysis also revealed a detectable increase in cleaved caspase-3 by ~3 days of mutant protein expression (Figure 7C). To identify the relative percentage of apoptotic cells in the mutant insulin expressing population we monitored apoptosis by TUNEL assay using FACS analysis, which also allowed for the detection of mutant protein expression by the GFP fluorescence. Addition of doxycycline increased GFP fluorescence depicted on the y-axis, compared to control cells not treated with doxycycline (Figure 7D). The percentage of apoptotic (TUNEL positive) cells is low and comparable to control cells following 24 h doxycycline induction (Figure 7D, left panels). However, mutant protein expression following 5 days of doxycycline treatment leads to the induction of apoptosis in approximately 14% of the population (Figure 7D, right panel).

**Figure 8:**
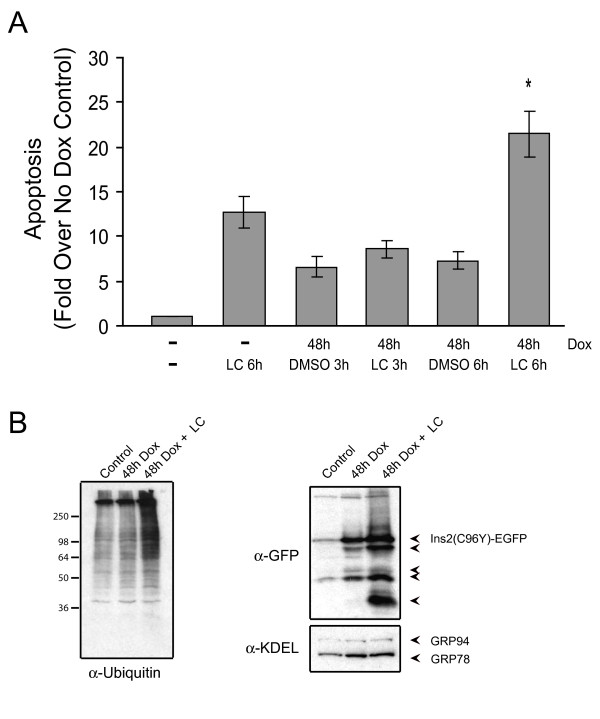
**The proteasome inhibitor lactacystin increases susceptibility of the mutant insulin expressing clone to apoptosis**. **A**. Clone #4S2 cells were untreated or treated with 2 μg/ml doxycycline for 48 h. Cells were then untreated or treated with 10 μM of lactacystin (LC) or an equivalent volume of DMSO for the times indicated. Apoptosis was measured using a cell death detection ELISA kit as described in the Methods. Shown are the mean ± SE of 3 independent experiments. *p < 0.05. **B**. Following 42 h of doxycycline treatment, cells were untreated, or treated with 10 μM of lactacystin (LC) or an equivalent volume of DMSO for 6 h in the presence of doxycycline. The cells were washed in PBS and lysed. Cell lysates (left panel) or TX-100 insoluble material (right panel) were resolved by SDS-PAGE and immunoblotted using the indicated antibodies. Note the increase in the levels of degradation products detected by the GFP antibody with LC treatment (arrowheads).

The extent of apoptosis observed in the population expressing Ins2 (C96Y)-EGFP even for extended periods (>5 days) seemed rather low. We hypothesized that the ER stress response in these cells, particularly the induction of ERAD genes, might be responsible for maintaining cell survival by degrading the misfolded insulin and preventing accumulation to toxic levels. We therefore examined whether inhibiting the ERAD system might sensitize the cells to cell death. As a first approach we inhibited proteasome function using lactacystin. Cells were induced with doxycycline for 48 h then treated or not with lactacystin and cell apoptosis was monitored. Inhibition of the proteasome resulted in a significantly higher percentage of apoptotic cells in the population (Figure 8A). This was also accompanied by an increase in the cellular levels of ubiquinated proteins (Figure 8B, **left panel**) and an increase in the levels of Ins2 (C96Y)-EGFP fusion protein and degradation fragments (Figure 8B, **right panel**). These results suggest that inhibiting ERAD increases misfolded protein levels and enhances ER stress-induced apoptosis.

**Figure 9:**
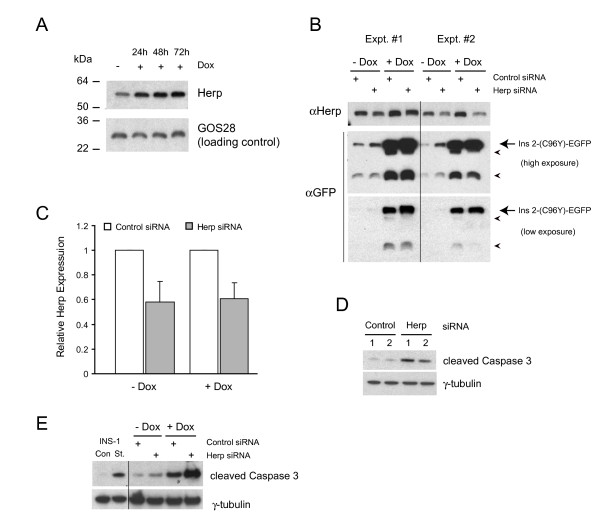
**Inhibiting Herp expression increases insulin 2 (C96Y)-EGFP levels and enhances apoptosis**. **A**. Clone #4S2 cells were untreated or treated with 2 μg/ml doxycycline for the times indicated and cell lysates were prepared and immunoblotted. **B**. Clone #4S2 cells were transfected with control siRNA or an siRNA directed against Herp and treated or not with doxycycline for 48 h. Cells were lysed and immunoblotted as indicated. A low and high exposure of the anti-GFP western blot is shown. Results from two of four independent experiments are shown. **C**. Immunoblots were quantified and the mean relative expression of Herp from 4 independent experiments is presented. **D**. Clone #4S2 cells were transfected with control or Herp siRNA and 48 h later apoptosis was measured by immunobloting for cleaved caspase 3. Results from two experiments are shown. **E**. Clone #4S2 cells were transfected as in (D) and 24 h later doxycycline was added or not for 48 h and apoptosis was measured by immunobloting for cleaved caspase 3. INS-1 cells treated or not with 0.3 mM straurosporine (St.) was included as a positive control for cleaved caspase 3.

As an alternative approach for inhibiting ERAD we targeted the Herp protein which was found to be induced early by mutant insulin expression (Table 1). Herp has been implicated in ERAD of certain misfolded proteins [23], although whether it is required for misfolded insulin degradation has not been examined. We first confirmed that the Herp protein is induced in response to mutant insulin expression (Figure 9A). To perturb ERAD function, we blunted the induction of Herp expression using siRNA (Figure 9B, C). Reducing Herp expression by about 40-50% resulted in an increase in the steady-state levels of the mutant insulin protein, even in the absence of doxycycline induction (Figure 9B). This also increased apoptosis levels in the population relative to control siRNA transfected cells (Figure 9D). With doxycyline induction for 48 h the levels of Ins2 (C96Y)-EGFP in Herp siRNA-treated cells was increased relative to control siRNA transfected cells, but the effect was small (Figure 9B). In some experiments we also observed a reduction in the degradation fragments in Herp siRNA-treated cells (see Figure 9B, **Expt.#2**, low exposure), but this was not a consistent finding (Figure 9B, **Expt.#1**). The inconsistent results may be due to the inefficient knock-down of the Herp protein achieved in these studies. Regardless, the levels of apoptosis in the population depleted of Herp expression was significantly higher compared to control siRNA-transfected cells as measured by cleaved caspase 3 levels (Figure 9E). Altogether, these results suggest that the mutant insulin is an ERAD target and that Herp function is required for its degradation and that reducing Herp levels increases mutant insulin levels and sensitizes the cells to apoptosis.

## Discussion

The UPR is an essential process by which cells respond to the accumulation of misfolded protein in the ER. This can occur either physiologically, as a result of acute secretory protein load, or as a result of pathological insults that may impede protein folding capacity. The basic ER stress response is well established involving an acute translational attenuation followed by the upregulation of a transcriptional profile that includes ER chaperone genes, ERAD components, protein transport genes, among others [2]. However, it is probable that different cell types have a unique profile of upregulated genes that are dependent on a particular cells function and the secretory proteins it produces. The goal of the present study was to examine the ER stress response in the insulin producing pancreatic β-cell. To do this we developed a pancreatic β-cell culture model with inducible expression of a folding-deficient insulin fusion protein based on the proinsulin mutation found in the Akita mouse [9]. The rationale behind this approach was that regulated expression of a single misfolded protein would allow for a temporal characterization of the UPR in β-cells. This may reflect the ER stress response to physiological conditions (acute insulin synthesis) and potentially a chronic response induced by pathological conditions occurring in type 2 diabetes (chronic insulin biosynthetic demands coupled with the effects of chronic free fatty acids, glucose and cytokines that induce ER stress).

Using the tetracycline/doxycycline-regulated expression system we created a double stable INS-1 cell clone with stable integration of the pTet-ON regulatory plasmid and the Ins2 (C96Y)-EGFP plasmid driven by the Tet/Dox-responsive promoter. The EGFP tag was used in order to facilitate selection of stable clones, as well as for sorting of the positive clones for cells with high expression of the Ins2 (C96Y)-EGFP fusion protein. In the clone selected for the studies basal expression of the mutant insulin is low, although detectable. Following doxycyline addition the mutant insulin is markedly induced by 24 h, reaching maximal expression by ~72 h and expression is maintained for several days. Expression of the mutant insulin fusion protein leads to a swollen ER lumen that is readily detectable in many cells and is indicative of misfolded protein accumulation and a stressed ER. This result is similar to the phenotype observed in the β-cells of Akita mice [9]. As expected, expression of folding-deficient Ins 2 (C96Y)-EGFP fusion protein resulted in the induction of UPR pathways (PERK, IRE1 and ATF6).

To analyze the ER stress response in the cell line we performed microarray expression profiling and real time PCR analysis at various times following mutant protein expression. We chose time-points of Ins2 (C96Y)-EGFP expression when no significant apoptosis was observed (24 h) and longer expression time points (48 h and 5 days), when apoptotic cell death was detectable. After 24 h, 45 genes were induced, while the number of induced genes was substantially increased after 72 h (86) and 5 days (68). Interestingly, the number of downregulated genes was also increased at the longer incubation times (37 and 56 after 48 h and 5 d of doxycycline induction, respectively). This may reflect the observation that prolonged activation of IRE1α can result in relaxed specificity and cleavage of cellular mRNAs [24], resulting in the downregulation of some genes as a result of prolonged ER stress.

After 24 h of Ins2 (C96Y)-EGFP expression the most consistently induced genes were ER resident chaperones and co-chaperones (*Grp78*, *Erdj4*, *P58*^*IPK *^, *Erdj3*, *Fkbp11*) and an ERAD component (*Herp*). The J-domain containing co-chaperones (*Erdj4*, *P58*^*IPK *^) have been shown to be induced by ER stress in various cell types [20,25-32]. Recently, P58^IPK ^and ERdj3 have been found to interact with misfolded proteins in the ER prior to recruitment of the chaperone GRP78 [26,29,30,32]. In addition, these proteins have also been implicated in targeting misfolded proteins for degradation via the ERAD system [20,28]. Thus, expression of misfolded insulin causes the induction of chaperone proteins early in an effort to fold the mutant insulin. These chaperones are maintained at high levels throughout the time course of mutant insulin induction and some may assist in degradation of the misfolded molecule once folding is found to be futile (discussed below). The importance of P58^IPK ^function in normal pancreatic β-cells *in vivo *is evidenced by increased pancreatic β-cell apoptosis and hyperglycemia in knock-out mice [33]. However, the detailed molecular function of these co-chaperones in β-cells has not been examined to date.

Interestingly, although there are six mammalian DNAJ domain-containing proteins, we only found a subset to be induced by mutant insulin expression. Notable among those that were not upregulated is ERdj5. Recently, expression of a folding-deficient version of the secreted protein surfactant protein C in HEK293 cells caused the induction of various ER stress response genes, including *Erdj4 *and *Erdj5*, which were shown to be required for degradation of the folding-deficient surfactant protein C [20]. Surfactant protein C, like proinsulin, contains disulfide bonds, but is not glycosylated. Thus, it is interesting that we do not observe induction of *Erdj5 *in β-cells expressing folding-deficient proinsulin. A recent report, however, has shown that ERdj5 can interact with EDEM proteins and is required for reducing disulfide bonds on misfolded, glycosylated proteins prior to retrotranslocation [21]. *EDEM *genes were not detected in the microarray analysis, suggesting that proinsulin may not require EDEM or ERdj5 for its degradation.

Comparing the UPR response observed in our study to ER stress responses in other cell types shows that some of the early chaperone genes induced by mutant protein expression are not observed in other systems. For example, tunicamycin treatment of neuroblastoma cells [34] or expression of a retroviral protein in astrocytes, which induces ER stress [35], did not cause an upregulation of *Erdj4*, *P58*^*IPK *^, *Erdj3*, or *Fkbp11*. Importantly, all of these genes have been shown to be induced by ER stress caused by prolonged palmitate exposure in cultured mouse MIN6 cells [36]. Saturated FFAs have been shown to induce ER stress in pancreatic β-cells and this may contribute to β-cell dysfunction [15,36,37]. In addition, a recent proteomic study examining islets from a diabetic insulin resistant mouse model has shown that the protein (and mRNA levels) of some of these (P58^IPK ^, ERdj3, Fkbp11) are upregulated [38]. Thus, the induction of these genes/proteins are likely important cell protective ER stress response proteins in β-cells and a detailed biochemical analysis of their function in mediating proinsulin folding and/or degradation is warranted.

In addition to ER chaperone genes and ERAD components, several other genes were found to be induced early as a result of Ins2 (C96Y)-EGFP expression, including *Sdf2l1*, *Armet *and *Creld2*. All three were abundantly upregulated at both early and late time points. The exact functions of the protein products of these genes in the ER stress response is largely undefined, although these proteins have also been reported to be induced by ER stress-inducing compounds such as tunicamycin in other cell types [39-41].

The appearance of prominent lower migrating bands recognized by anti-GFP antibodies indicates that the Ins2 (C96Y)-EGFP fusion protein is being degraded. Similar cleavage products have been detected with wild-type proinsulin tagged with GFP at the C-terminus and was suggested to occur from non-specific cleavage of the fusion protein at some point along the secretory pathway [42]. In the case of the Ins2 (C96Y)-EGFP the cleavage products are not efficiently immunoprecipitated by anti-GFP antibodies and are not detected in ER fractions. Thus, the smaller migrating bands recognized by GFP antibodies are likely degradation products of proteolysis occurring in the cytoplasm. The full-length mutant insulin fusion protein appears to be inaccessible to the secretory pathway and is retained in the ER, which is consistent with a report showing that the C96Y mutation in proinsulin results in misfolding of the molecule and retention in the ER [42].

The Ins2 (C96Y)-EGFP fusion protein is likely degraded by the ubiquitin-dependent ERAD system. The ERAD-associated genes *Herp *and *Sel1 *were induced by mutant insulin expression, which is consistent with the finding that *Sel1 *is upregulated in Akita islets [43]. Inhibiting the 26S proteasome resulted in an increase in the levels of Ins2 (C96Y)-EGFP and sensitized the cells to cell death. This was also the effect observed by reducing Herp expression using siRNA, a protein recently shown to be required for the degradation of a disulfide bond-containing mutant version of the kappa light chain [23]. The process of Ins2 (C96Y)-EGFP degradation however, may be quite complex. If the proteasome was responsible for generating the degradation fragments observed (Figure 2) than inhibiting the proteasome would be expected to not affect the steady-state levels of these fragments; new fragments would be prevented from being generated from Ins2 (C96Y)-EGFP and fragments already present prevented from being degraded. However, most degradation fragments were actually increased by proteasome inhibition (Figure 8B), suggesting that other proteases may be responsible for generating the degradation fragments that are then targeted for ubiquitin-dependent degradation.

It should be pointed out that although expression of the Ins2 (C96Y)-EGFP fusion protein induces ER stress and degradation of the fusion protein, due to the presence of the EGFP tag the degradation of the fusion construct may not be identical to the degradation of untagged proinsulin C96Y. Furthermore, it is possible that the ER stress response elicited by untagged proinsulin C96Y is different compared to that of Ins2 (C96Y)-EGFP. These possibilities should be explored in future studies.

This model system also allowed us to examine potential gene expression changes associated with ER stress-induced cell death. An upregulation of the pro-apoptotic transcription factor *Chop *and one of its target genes (*Trib3*) was evident by 24 h of Ins2 (C96Y)-EGFP expression, which is indicative of PERK pathway activation and is likely to be a normal physiological response. At this time point no significant apoptosis is detected in the population. After 48 h of mutant insulin expression however, the levels of *Chop *and *Trib3 *were even higher, which coincided with detection of apoptosis in the population. Thus, a sustained increase in the levels of CHOP and TRIB3 proteins may tilt cells towards apoptosis induction. Pancreatic β-cells of CHOP-deficient heterozygous Akita mice are partially protected from cell death [12], although clearly there are other factors also involved. In the mutant insulin expressing cell line the most highly induced gene after 48 h was *Trib3*. Recently, the TRIB3 protein has been shown to be induced by the PERK-ATF4-CHOP pathway and has been implicated in mediating apoptosis [18,19] and in hyperglycemia-induced pancreatic β-cell death [44]. The mechanism of this effect, however, is unclear. Whether the TRIB3 protein is responsible for apoptosis induction and how much of the apoptosis is TRIB3-dependent in this model system requires future study.

Despite the abundant upregulation of *Chop *and *Trib3 *in response to mutant insulin expression, the level of apoptotic cells in the population remained rather low, even after long term expression of the mutant construct. This is likely because one of the main cell protective effects induced by the UPR, ERAD, is also upregulated. Inhibition of ERAD by inhibiting the cytosolic proteasome or inhibiting Herp upregulation sensitizes the cells to apoptosis, supporting the notion that the ERAD pathway may support cell survival by degradation of misfolded insulin.

## Conclusions

The β-cell model of ER stress described in this study has allowed for a detailed characterization of the ER stress response induced by misfolded Ins2 (C96Y)-EGFP expression and for the identification of both cell protective and possible cell destructive genes. The repertoire of genes/proteins induced is unique to this cell type compared to the UPR induced in other cell types, perhaps reflecting the unique role of β-cells to produce insulin. The Herp protein and ER-associated degradation is essential for maintaining cell survival in cells expressing mutant insulin. The cell model developed here will be useful in studies examining the function of ER stress-induced genes.

## Competing interests

The authors declare that they have no competing interests.

## Authors' contributions

TH was responsible for apoptosis analysis and the real-time PCR and microarray studies. EL was responsible for generating the mutant insulin expressing cell line and some of the studies examining the ER stress response. MS contributed experiments reported in Figure 9. TT and LZ contributed experiments reported in Figure 4. All authors made substantial contributions to the design of the experiments, acquisition and analysis of data and preparation of the figures. AV supervised these studies and prepared the manuscript. All authors read and approved the final manuscript.

## Supplementary Material

Additional file 1 **This file lists all the genes whose expressed increased or decreased >2-fold in at least two of three independent experiments**. Excel file presenting all of the Affymetrix probes whose expression was increased or decreased by at least 2 fold between doxycycline-treated vs. control, untreated INS-1 clone #4S2 cells. Data from 3 independent experiments (N = 1, N = 2, N = 3) for all doxycycline treatment time points (24 h, 48 h, and 5 days) is shown. Note that gene descriptions for these tables are provided by the GeneSpring software. Bold indicates probes whose expression was changed in all three experiments, while regular font indicates genes that were changed in at least 2 of 3 independent experiments. The primary data files for the microarray experiments have been deposited in the Gene Expression Omnibus (GEO) database (GSE22537). Click here for file

Additional file 2 **This file lists the well substantiated genes whose expressed increased or decreased >2-fold in at least two of three independent experiments**. Excel file presents expression data for well substantiated genes derived from the data presented in additional file 1. The putative function of the genes is derived from the NCBI database. Data from 3 independent experiments for all doxycycline treatment time points is provided. Click here for file
